# A Comparison of Substrate Utilization Profiles During Maximal and Submaximal Exercise Tests in Athletes

**DOI:** 10.3389/fpsyg.2022.854451

**Published:** 2022-04-08

**Authors:** Rohit Ramadoss, Joseph R. Stanzione, Stella Lucia Volpe

**Affiliations:** ^1^Department of Human Nutrition, Foods, and Exercise, Virginia Polytechnic Institute and State University, Blacksburg, VA, United States; ^2^Worldwide Sport Nutritional Supplements, Inc., Bohemia, NY, United States

**Keywords:** combat athletes, runners, substrate utilization, fat, carbohydrate

## Abstract

**Background:**

Exercise is primarily sustained by energy derived from lipids (plasma free fatty acids and intramuscular triglycerides), and glucose (plasma glucose and muscle glycogen). Substrate utilization is the pattern by which these fuel sources are used during activity. There are many factors that influence substrate utilization. We aim to delineate the effect of exercise intensity and body composition on substrate utilization.

**Objective:**

The objective of our study was to discern the differences in substrate utilization profiles during a maximal and submaximal graded exercise test, and to determine the extent to which body composition influences substrate utilization during the exercise tests.

**Methods:**

A total of 27 male athletes, 32.5 ± 11 years of age, were recruited for this study. Body composition was analyzed using a bioelectrical impedance analyzer. Maximal and submaximal exercise tests were performed on a treadmill. A novel graded submaximal treadmill protocol was used for the submaximal test.

**Results:**

Average percent body fat (PBF) was 15.8 ± 5%. Average maximal oxygen consumption (VO_2_max) was 47.6 ± 9 mL/kg/min, while the average exercise intensity (percent VO_2_max) at which participants were shifting to glucose predominance for energy during the maximal and submaximal tests were 76 ± 8.3% and 58.4 ± 21.1%, respectively. A paired-samples *t*-test was conducted to compare percent VO_2_max at crossover point in maximal and submaximal graded exercise tests. There was a significant difference in percent VO_2_max at the crossover point for maximal (76 ± 8.3%) and submaximal (58 ± 21.1%) tests (*t* = 4.752, *p* = 0.001). A linear regression was performed to elucidate the interaction between exercise intensity at the crossover point and body composition during a maximal and submaximal graded exercise test. There was a significant effect of PBF on percent VO_2_max at crossover point during the maximal graded exercise test [*F*(1,24) = 9.10, *P* = 0.006] with an R^2^ of 0.245. However, there was no significant effect of PBF on percent VO_2_max at crossover point during the submaximal graded exercise test (*P* > 0.05).

**Conclusion:**

Substrate utilization, represented by the crossover point, is dependent on the rate of increase in exercise intensity. At maximal efforts, the crossover to carbohydrates from fats as the predominant fuel source occurs at a significantly later stage of percent VO_2_max than at submaximal efforts. Furthermore, body composition represented by PBF is a significant predictor of substrate utilization during maximal efforts. Athletes with a relatively higher PBF are more likely to have increased lipid oxidation during high intensity exercises than those with a lower body fat percentage.

## Introduction

Glucose (plasma glucose and muscle glycogen) and fats (plasma free fatty acids and intramuscular triglycerides) are the primary sources of energy during any form of physical exertion (Spriet and Watt, 2003). Substrate utilization is an area of growing interest and research.

The term “substrate utilization,” represents the contribution of fat and carbohydrates to energy expenditure during exercise. The proportion of their contribution during energy expenditure is modulated by several factors, including intensity and duration of exercise, age, training status, sport specificity, diet, gender and body composition. However, the primary determinant of substrate utilization is exercise intensity (Fogelholm, 2006). Exercise intensity, which is expressed as a percentage of the maximal oxygen consumption (VO_2_max), can be defined as the amount of physical power generated by the body while performing a physical activity. During low exercise intensities (<60% VO_2_max), most of the energy requirement is met with the oxidation of fatty acids, of which more than 85% is derived from the plasma (Brooks, 1997). As the exercise intensity increases to a moderate level (65% VO_2_max), availability of plasma free fatty acids decreases while intramuscular triglyceride oxidation increases (Lanzi et al., 2014). At moderate exercise intensities, plasma free fatty acids and intramuscular triglycerides contribute equally to total fat oxidation, and overall fat oxidation tends to be the highest at this level of exercise intensity (Lanzi et al., 2014). At higher exercise intensity levels (>85% of VO_2_max), the rate of plasma free fatty acid oxidation is substantially decreased relative to moderate intensities. Moreover, intramuscular triglycerides become the major source of fatty acids for oxidation. However, at higher exercise intensities, the absolute rate of fat oxidation decreases, while the oxidation of glucose contributes to more than two-thirds of the energy required (Kriketos et al., 2000; Lanzi et al., 2014). This transition from free fatty acid oxidation to glucose oxidation during high intensity exercise stems from glucose’s capacity to provide energy at a faster rate compared to free fatty acids (Pérez-Martin et al., 2001). In other words, relative to glucose oxidation, fatty acid oxidation has a limited capacity per unit time in generating adenosine triphosphate (ATP). Therefore, during low intensity exercise, fat is the predominant energy substrate due to its relatively higher abundance in the body, whereas during high intensity exercise, glucose is the major contributor to energy requirements.

Another key concept that is relevant to the study of substrate utilization is the “crossover point.” The crossover point represents the intensity at which oxidation of glucose overtakes fatty acids as the predominant fuel source during exercise, and thus, serves as a marker of metabolic flexibility (Brooks, 1997). In our study, we utilized the crossover point as a proxy marker to represent the moment of exercise intensity at which athletes shifted to predominantly oxidizing glucose from fatty acids for their energy needs.

Furthermore, there has been evidence concerning the effect of body composition on fat oxidation rates during exercise (Kriketos et al., 2000; Pérez-Martin et al., 2001; Lanzi et al., 2014). Maximal fat oxidation rates were found to be lower in individuals with higher levels of adiposity compared to leaner individuals (Taylor et al., 1955; Duncan et al., 1997; Yoon et al., 2007). However, most researchers who have observed this difference have done so by comparing overweight individuals with a control group within the healthy weight range. Although these are important studies on the effect of body composition on substrate utilization, there is limited research to suggest a similar effect of body composition on substrate utilization within a group of athletes.

The rationale for our study is to further increase the understanding of substrate oxidation in athletes as it pertains to factors such as rate of increase in exercise intensity and body composition. Prior literature has substantiated the role of both exercise intensity and body composition. However, the rate of change of exercise intensity (e.g., how quickly exercise intensity increases), has not been extensively investigated. Moreover, research relating body composition to substrate utilization patterns have not been conducted in an athletic sample. As such, we aimed to quantitatively characterize and compare the differences in substrate utilization based on rate of change of exercise intensity and body composition during maximal and submaximal graded exercise tests in male athletes, 18 years of age and older. The first hypothesis of our study was that there would be a difference in percent VO_2_max at the crossover point based on the rate of change of exercise intensity. Our second hypothesis was that there would be a difference in percent VO_2_max at the crossover point based on body fat percentage. Specifically, we hypothesized that individuals with a higher body fat percentage will shift to glucose oxidation predominance later than individuals with a relatively lower body fat percentage.

## Methods and Procedures

### Participants

Male athletes, 18 years of age and older, where recruited for this study. All participants had more than 3 years of experience in their respective sport, which included running and grappling (Wrestling, Jiu-jitsu, and Judo). All participants read and signed an approved informed consent document. The study was approved by the Institutional Review Board.

### Preliminary Measurements

During the first visit, upon the completion of informed consent, we collected demographic information pertaining to age, gender, sporting background and training history. Participants completed a food frequency questionnaire (FFQ) (2005 Block Food Frequency Questionnaire Version 1, NutritionQuest, Berkeley, CA, United States) that reflected their dietary habits over the previous year to the date of their visit. The FFQ estimates usual and customary intake of a wide array of nutrients and food groups comprising approximately 110 food items. The list of foods was developed from the National Health and Nutrition Examination Survey (NHANES) 1999–2002 dietary recall data, while the nutrient database was developed from the United States Department of Agriculture (USDA) Food and Nutrient Database for Dietary Studies (FNDDS; NutritionQuest). Food frequency questionnaires have been studied to be a reliable method of documenting food intake among different populations (Paul et al., 2005; Fernández-Ballart et al., 2010). The Block FFQ has been shown to have a moderate to high validity in capturing dietary intake pattern for up to 1 year (Boucher et al., 2006).

### Measurement of Anthropometry and Body Composition

Height (in centimeters) was measured twice using a stadiometer. Body weight was measured using a calibrated Seca 700 balance beam scale (Seca, Hamburg, Germany). The average of each of the two measures was used for analyses. Multiple measurements were taken to ensure accuracy. Body composition was measured using InBody 520 (InBody USA, Cerritos, CA, United States) bioelectrical impedance analysis (BIA) scale.

### Experimental Protocol

The experimental protocol consisted of two visits to our lab on separate occasions. During the first visit, the Maximal Graded Exercise Test (MGET) was conducted, while on the second visit the Submaximal Graded Exercise Test (SGET) was conducted. Each of these exercise tests were conducted on a standardized treadmill where the participant would exercise while their volume of the respiratory gases expired during exercise was continuously measured throughout the duration of the test. These exercise tests were performed to measure VO_2_max and study substrate utilization [respiratory exchange ratio (RER)] during exercise (Taylor et al., 1955; Duncan et al., 1997; Astorino et al., 2000; Yoon et al., 2007). Measurements during both tests were assessed using indirect calorimetry (Oxycon™ Mobile Device, Vyaire™ Medical, Yorba Linda, CA, United States) and a laptop operating Jaeger JLab version 5.3x (2010) software to analyze the volume of inspired and expired air. The Oxycon mobile unit was placed on the participant’s body using a comfortable vest, which held the device on their back such as not to hinder any form of physical movement. A mask, housing a flat fan system near the mouth, was fitted onto the participant’s face and secured around the back of their head. The indirect calorimetry device records breath-by-breath data of the volume of respiratory exchange during physical activity using a patented TripleV Volume sensor (Vyaire Medical CareFusion Oxycon Mobile IFU). In a study where the efficacy of several indirect calorimetry instruments were compared, the Oxycon was found to have a variance of <3% from the true value (Carter and Jeukendrup, 2002; Indirect Calorimetry, 2017). In addition to its accuracy, the Oxycon mobile unit also allows for increased mobility due to its compact size. A chest-positioned Polar Electro (Kempele, Finland) heart rate monitor was also worn during the test to measure heart rate. Participants abstained from any exercise, caffeine and alcohol for 12 h prior to each testing session.

### Maximal Graded Exercise Test

The MGET was designed to determine VO_2_max by systematically increasing the exercise intensity *via* increments in incline and/or speed. The MGET was conducted using a modified Taylor protocol (Taylor et al., 1955). The modified Taylor protocol began with a warm-up phase of 2 min at 7 miles per hour (mph) at 0% incline. Following the warm-up phase, the test phase began at 7 mph for the first minute. At the end of every minute, the incline gradient was increased by 1% and continued to increase until volitional exhaustion. If an incline of 12% gradient was attained, the incline remained at 12% while the speed of the treadmill increased by 0.5 mph every minute until the participant signaled exhaustion. Upon exhaustion, the testing protocol entered the cool-down phase for 2 min at 3.5 mph at 0% incline. During the entirety of the exercise test, breath-by-breath oxygen consumption and carbon dioxide production were measured continuously through the indirect calorimeter. To evaluate that a true VO_2_max had been achieved, we ensured that there was a flattening of oxygen consumption with an increased workload. In addition, maximum heart rate had to be near the maximum age-predicted heart rate (±5 bpm from maximum heart rate), and the RER had to be >1.1.

### Submaximal Graded Exercise Test

The submaximal graded exercise test (SGET) utilized each participant’s predefined VO_2_max determined from the MGET to tailor the intensity of the test at the correct percent VO_2_max values for each participant. The SGET protocol consisted of a warm-up phase of 5 min at 3.5 mph at 0% incline. Following this, the test phase began by increasing the speed by 0.5 mph every 30 s until the participant reached 35% of his VO_2_max. Upon reaching 35% VO_2_max, the participants exercised at this intensity for 3 min. After 3 min, the speed was increased by 0.5 mph every 30 s until 50% VO_2_max was reached. Upon reaching 50% VO_2_max, the participants exercised at this intensity for 3 min. We used the same protocol to achieve 65, 80, and 95% of VO_2_max, and participants exercised at each intensity for 3 min. The test phase was concluded when the participant reached a RER value >0.92, indicating predominant utilization of carbohydrates for energy. The cool-down phase began following the test phase, where participants exercised for 2 min at 3.5 mph at 0% incline. This graded exercise test was a modified version of a validated test used in prior studies (Achten et al., 2002; Achten and Jeukendrup, 2004b; Venables et al., 2005).

### Statistical Analyses

A power analysis was conducted with G*Power prior to the study (Faul et al., 2009). A total sample size of 26 was calculated using an effect size of 0.33, an alpha error rate of 0.05, and a power of 0.8. Mean, standard error of the mean (SEM), and range was calculated for each variable. A paired-samples *t*-test was conducted to compare percent VO_2_max at crossover point in maximal and submaximal graded exercise tests. This test was used to determine if there was a significant difference in the exercise intensity at which participants were crossing over to glucose oxidation between the two exercise tests. Linear regression was calculated to predict percent VO_2_max at the crossover point based on percent body fat during maximal and submaximal graded exercise tests. This test was utilized to measure the effect of body fat percentage on the crossover point during the two exercise tests (e.g., the reliability of body fat percentage at predicting the metabolic shift to glucose predominance during exercise). For all statistical analyses, the alpha value was set *a priori* at 0.05. All statistical analyses were conducted using IBM SPSS Statistics for Windows, Version 26.0 (IBM Corp., Armonk, NY, United States).

## Results

### Participant Description

Table 1 depicts the descriptive characteristics of the study population. We intentionally chose participants who had an experience of three or more years in their sporting background (running or grappling). As such, all participants can be classified as athletes. Percent body fat, body mass index (BMI), and VO_2_max were excellent, due to the fact our participants were all athletes. However, there was a range in these values due to the two types of sports represented, and the wide age range of the athletes in our study.

**TABLE 1:** Descriptive characteristics of the study population of runners (*n* = 18) and grapplers (*n* = 8).

Descriptive variable	Mean ± SD	Range (minimum to maximum)
Age (years)	32.5 ± 11.5	20–67
Body weight (kg)	75.4 ± 7.8	59–98
VO_2_max (mL/kg/min)	47.7 ± 8.9	34.9–70.9

*SD, standard deviation; kg, kilograms; m, meters; BMI, body mass index; VO_2_max, maximal oxygen consumption; mL, milliliters; min, minute.*

### Differences in Crossover Point Between Maximal and Submaximal Graded Exercise Test

**FIGURE 1:**
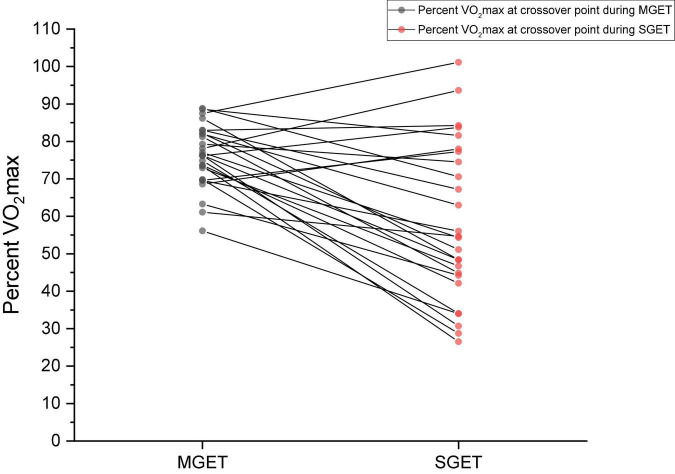
Individual comparison of percent maximal oxygen consumption (VO_2_max) at crossover point during the maximal graded exercise test (MGET) and submaximal graded exercise test (SGET).

The maximal graded exercise test was conducted on a treadmill using a modified Taylor protocol, while the submaximal graded exercise test was conducted using a novel treadmill protocol utilizing predetermined values from the MGET. Tables 2, 3 depict the variables from the maximal and submaximal graded exercise test, respectively. A paired-samples *t*-test was utilized to compare percent VO_2_max at crossover point in the MGET and SGET. There was a significant difference in percent VO_2_max at the crossover point for maximal (76 ± 8%) and submaximal (58 ± 21%) tests (*t* = 4.752, *p* = 0.001). Figure 1 depicts the comparison of percent VO_2_max at crossover point between the MGET and SGET. This indicates that participants were crossing over to predominant carbohydrate oxidation for fuel much later in MGET than in SGET, which suggests that the rate of change in intensity plays a role in substrate utilization during exercise.

**TABLE 2:** Outcome measures assessed during the maximal graded exercise test.

Outcome measures	Mean ± SD	Range (minimum to maximum)
VO_2_ at crossover point (mL/kg/min)	35.74 ± 4.6	25.7–44.6
Percent VO_2_max at crossover point (%)	76.1 ± 8.4	56.1–88.8
Heart rate at crossover point (bpm)	141.7 ± 11.9	117–161
Time to crossover point (Minutes)	5 ± 1.6	3–10.5

*SD, standard deviation; VO_2_, oxygen consumption; mL, milliliters; kg, kilograms; min, minute; VO_2_max, maximal oxygen consumption; bpm, beats per minute.*

**TABLE 3:** Outcome measures assessed during the submaximal graded exercise test.

Outcome measures	Mean ± SD	Range (minimum to maximum)
VO_2_ at crossover point (mL/kg/min)	27.1 ± 9.1	12.7–51.3
Percent VO_2_max at crossover point	58.5 ± 21.1	26.5–99.14
Heart rate at crossover point (BPM)	120.3 ± 25.7	81–161
Time to crossover point (Minutes)	15.2 ± 6.5	6–27

*SD, standard deviation; VO_2_, oxygen consumption; mL, milliliters; kg, kilograms; min, minute; VO_2_max, maximal oxygen consumption; bpm, beats per minute.*

### Body Composition and Substrate Utilization

**FIGURE 2:**
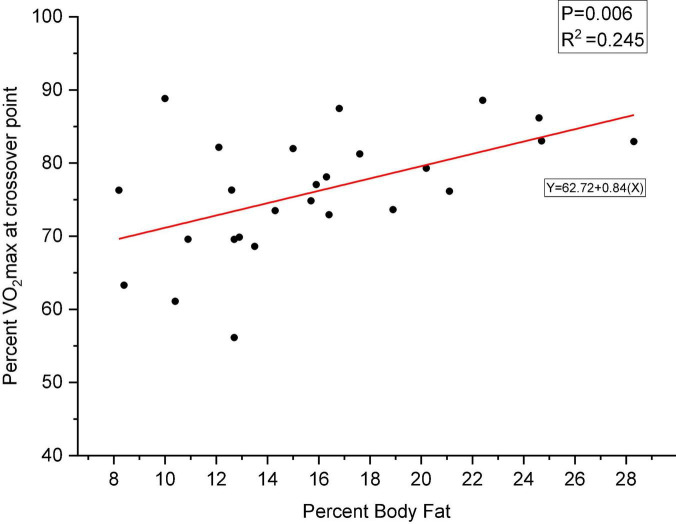
Relationship between percent maximal oxygen consumption (VO_2_max) at crossover point and percent body fat during the maximal graded exercise test (MGET) established by linear regression. *P* = 0.006, significant relationship between a higher percent body fat and lipid utilization as the primary substrate (e.g., delays crossover to using carbohydrate). The red line is the regression line expressing the hypothesized relationship between percent VO_2_max at crossover point and percent body fat.

**FIGURE 3:**
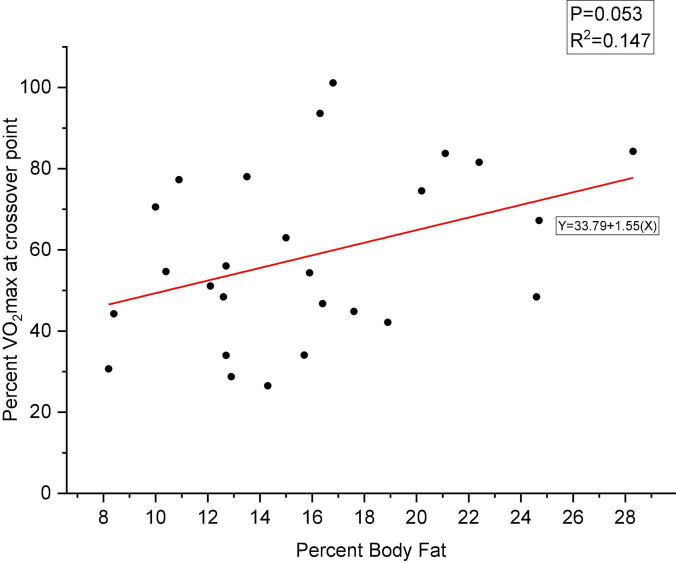
Relationship between percent maximal oxygen consumption (VO_2_max) at crossover point and percent body fat during the submaximal graded exercise test (SGET). *P* = 0.053, there was a margin of statistical significance between a higher percent body fat and lipid utilization as the primary substrate (e.g., delays crossover to using carbohydrate). The red line is the regression line expressing the hypothesized relationship between percent VO_2_max at crossover point and percent body fat.

Body composition was represented using percent body fat (PBF) measured with a BIA, while substrate utilization was determined using RER derived from the measurements of volumetric gas exchange ratio using the indirect calorimeter while exercising. A RER > 0.85 illustrates a carbohydrate oxidation predominance, while an RER < 0.85 indicates lipid oxidation predominance for energy. A linear regression was calculated to predict percent VO_2_max at crossover point based on PBF during the maximal and submaximal tests. There was a significant effect of PBF on percent VO_2_max at crossover point during the maximal graded exercise test [*F*(1,24) = 9.10, *P* = 0.006] with an R^2^ of 0.245. However, although approaching significance, there was no significant effect of PBF on percent VO_2_max at crossover point during the SGET (*P* = 0.053). Figure 2 depicts the effect of PBF on percent VO_2_max at crossover point during the MGET. Figure 3 depicts the effect of PBF on percent VO_2_max at crossover point during the SGET.

## Discussion

There are four major contributors to energy during physical exertion, namely, plasma glucose, muscle glycogen, plasma free fatty acids, and intramuscular triglycerides. The amount of energy obtained from the oxidation of each of these contributors varies based on many factors. One of the primary drivers of substrate utilization is exercise intensity. This is due to inherent differences in how rapidly energy can be obtained from oxidizing glucose relative to plasma free fatty acids. Plasma free fatty acids cannot be oxidized at high enough rates to provide all the energy required for moderate to high intensity exercise. This observation has been consistently reported by many researchers who compared substrate oxidation rates at different exercise intensities (Coyle, 1995; Treuth et al., 1996; Achten et al., 2002; Berger, 2004; Venables et al., 2005). The energy for exercise at low intensities (<45% VO_2_max) is primarily derived from plasma free fatty acids and intramuscular triglycerides, while the energy for high intensity exercise (approximately >85% VO_2_max) is primarily obtained from plasma glucose and muscle glycogen. However, substrate utilization is a complex phenomenon with many drivers. Substrate utilization at rest and during exercise is mediated by acute and chronic dietary patterns (Hellerstein, 1999; Gregory et al., 2011), gender (Chenevière et al., 2011), training status (Lima-Silva et al., 2010), modality of exercise (Achten and Jeukendrup, 2004a), ethnicity (Hickner et al., 2001), aerobic fitness (Venables et al., 2005), insulin resistance (Braun et al., 2004), menstrual phase (Zderic et al., 2001), fat deposition pattern (Venables et al., 2005), and circadian rhythm (Mohebbi and Azizi, 2011), among other factors.

With respect to the role of diet, it may be that consuming a carbohydrate meal prior to exercise affects substrate oxidation. Achten et al. (2003) conducted a crossover design study where they evaluated the effects 75 g of glucose compared to a placebo given 45 min prior to exercise in 11 moderately trained cyclists. The authors observed that, when the cyclists consumed glucose prior to exercise, their fat oxidation rate was significantly lower compared to the placebo, suggesting that acute dietary intake (Zderic et al., 2001) affects substrate utilization.

Chenevière et al. (2011) assessed the effect of gender on substrate oxidation patterns, and reported that women had higher levels of lipid oxidation compared to men at the same exercise intensity. It is hypothesized that the difference in lipid oxidation between women and men is predominantly driven by estrogen. Estrogen plays a role in many aspects of energy metabolism, such as insulin sensitivity and catecholamine-stimulated lipolysis. Moreover, lipid oxidation tends to be higher in women during the luteal phase of the menstrual cycle when circulating estrogen is high (Zderic et al., 2001).

Another factor that plays a role in lipid oxidation is training status. Venables et al. (2005) observed the difference in fat oxidation rates between moderately trained cyclists and highly trained cyclists during exercise. They reported that the highly trained cyclists had significantly higher fat oxidation rates compared to moderately trained cyclists at the same intensity, suggesting that training experience plays a role in fat oxidation.

Exercise modality also plays a role in substrate utilization. Privette et al. (2003) compared the difference in fat oxidation between running and cycling. They reported that running elicited a higher rate of fat oxidation than cycling at the same exercise intensity (Achten et al., 2003).

Race and ethnicity have been shown to influence substrate utilization. It has been reported that African Americans demonstrate reduced lipid oxidation at rest, during exercise, and when challenged with a high fat diet (Hickner et al., 2001; Privette et al., 2003).

Insulin resistance also plays a role in substrate utilization. Braun et al. (2004) reported that individuals with insulin resistance rely less on muscle glycogen and more on lipids during exercise compared to BMI-matched individuals without insulin resistance.

Although multiple factors play a role in dictating substrate oxidation, there is a general pattern of substrate oxidation as exercise intensity increases (Coyle, 1995). At low intensities of exercise, energy is primarily derived from lipid oxidation, while carbohydrate oxidation is minimal or negligible. As exercise intensity increases, a shift occurs between carbohydrate and lipid oxidation rates. Carbohydrate oxidation begins to increase and lipid oxidation declines as exercise intensity rises. There comes a point where energy is predominantly derived from carbohydrate oxidation overtaking lipid oxidation as the predominant energy source. This point is known as the crossover point (Brooks, 1997; Berger, 2004), and is represented using RER. The primary focus of our study was to observe the effect of rate of change in exercise intensity and body composition on substrate utilization patterns as represented by the crossover point in male athletes. Rate of change of exercise intensity, the time it takes for exercise intensity to incrementally rise, is not quite as extensively studied. We are yet to fully delineate the effect of time with respect to exercise intensity. For instance, what would the difference in substrate utilization pattern be if a 60% VO_2_max is reached in 5 min versus 15 min? As such, we aimed to find the difference in substrate utilization patterns between two exercise tests differing in how rapidly exercise intensity increases. Here, we compared the effect of rate of change in exercise intensity by utilizing a maximal exercise test, which has a higher rate of change in exercise intensity compared to a submaximal exercise test, which has a much lower rate of change in exercise intensity. Between the two tests, participants demonstrated switching to using predominantly glucose at a lower percent VO_2_max during the submaximal test compared to the maximal test. Therefore, when the time it takes for exercise intensity to increase is longer, the metabolic shift to utilizing plasma glucose and muscle glycogen for fuel is quicker. Thus, the crossover point occurs at a lower exercise intensity when the rate of change in exercise intensity is slower. By understanding the point at which a metabolic shift in fuel utilization occurs in high intensity and low intensity exercises, the practical implications of this finding may be useful in educating exercise paradigms that aim for optimizing lipid oxidation. This may include weight loss programs that incorporate an exercise component with lipid oxidation as a primary goal. Furthermore, it may also benefit endurance athletes in dictating their exercise intensities during training sessions.

With respect to body composition, several researchers (Lanzi et al., 2014; Santiworakul et al., 2014; Purdom et al., 2018; Perez et al., 2019) have suggested that overweight individuals demonstrate a higher predominance of lipid oxidation at similar exercise intensities compared to relatively leaner individuals. However, this phenomenon has not been widely reported in an athletic population. We aimed to understand the effect body composition (as represented by PBF) has on substrate utilization by observing the exercise intensity (percent VO2max at crossover point) at which participants switched to a carbohydrate predominance for energy (e.g., the crossover point). Linear regression analysis demonstrated a significant relationship between PBF and percent VO_2_max at crossover point during MGET but not during SGET. This suggests that, among the tested athlete population, those with a higher body fat percentage prolonged the metabolic switch to predominantly utilizing plasma glucose and muscle glycogen for energy. The significance of this finding may play a role in further elucidating the nuanced factors that drive substrate utilization. Nonetheless, the practical implications of this finding may benefit athletes to have an idea of where their substrate utilization shifts to glucose predominance depending on their unique body composition. Fatigue during intense prolonged exercise is commonly due to the depletion of muscle and liver glycogen (Coyle, 1995); therefore, it may be beneficial to prolong tapping into these resources until necessary. When equipped with knowledge about their unique crossover points, athletes will be able to preserve their glycogen stores and prolong fatigue by exercising within the threshold of lipid oxidation, and strategically plan their training sessions for meeting their performance goals.

There are several limitations to our study. The measurement of body composition through BIA is constrained by the hydration status of participants, which serves as a confounding factor, affecting the precision of percent body fat measures. The duration, sporting background, and type of training employed by our participants in our sample was varied. These varied aspects of their lifestyle are also potential confounding factors that affect the relationship between exercise intensity, body composition and substrate utilization. Finally, participants were tested during different times of the day, depending on their schedules. Thus, circadian rhythm on substrate oxidation is another potential confounding factor.

## Conclusion

We demonstrated the relationship between rate of change of exercise intensity, body composition and substrate utilization patterns represented by the crossover point. We found that both rate of change in intensity of exercise and body composition play a role in dictating substrate oxidation during exercise. When exercise intensity increases at a rapid pace, the crossover to glycogen and glucose utilization for energy is delayed when compared to increasing exercise intensity at a slower pace. Moreover, percent body fat is positively correlated to substrate utilization patterns. The switch to glucose from lipid oxidation happens at a higher intensity in individuals with higher percent body fat.

## Data Availability Statement

The raw data supporting the conclusions of this article will be made available by the authors, without undue reservation.

## Ethics Statement

The studies involving human participants were reviewed and approved by the Drexel University Institutional Review Board. The patients/participants provided their written informed consent to participate in this study.

## Author Contributions

SV contributed to the supervision and project administration and had the primary responsibility for reviewing and editing the final content. JS conceptualized the study, collected and analyzed the data, and performed the statistical analyses. RR helped to collect and analyzed the data and performed the statistical analyses. All authors commented and approved the final version of the document.

## Conflict of Interest

JS was employed by the Worldwide Sport Nutritional Supplements, Inc. The remaining authors declare that the research was conducted in the absence of any commercial or financial relationships that could be construed as a potential conflict of interest.

## Publisher’s Note

All claims expressed in this article are solely those of the authors and do not necessarily represent those of their affiliated organizations, or those of the publisher, the editors and the reviewers. Any product that may be evaluated in this article, or claim that may be made by its manufacturer, is not guaranteed or endorsed by the publisher.
